# Supplementary material for: Improving Audiology Student Training by Clinical Simulation of Tinnitus: A Glimpse of the Lived Experience of Tinnitus
Source: Brain Sci. 2023 Sep 17;13(9):1338. doi: 10.3390/brainsci13091338 (PMC10526917; doi:10.3390/brainsci13091338)
Supplement: Supplementary file 1 [file brainsci-13-01338-s001.zip › brainsci-2601931-supplementary.pdf]

Supplementary Material S1

Name of the student : ___________________

Team : ______________________

Date of laboratory : _______________________

Tinnitus Psychoacoustics Laboratory: Evaluation Sheet

1) Ask the patient to describe their tinnitus: **record it on your evaluation sheet. If the patient has difficulty describing his tinnitus, guide him through the process. Ask him questions on the form, pitch, intensity, laterality of their tinnitus senstation.**

2) In order to determine the timbre of tinnitus: present in the following order white noise, narrowband noise and a pure tone and ask the patient which of the 3 sounds resembles most their tinnitus sensation (note: the 3 possibilities can be used during the exercise, but not in combination). **Record it on your evaluation sheet.** *if it is White Noise do not do the frequency pairing, go to step 4

3) In order to determine the pitch of the tinnitus, present the previously determined sound centered at 1000 Hz, then the same sound one octave below or above. Repeat the forced choice comparisons until you achieve a faithful answer. **Record each response of the patient in your evaluation sheet.** (Note: for the exercise the person in charge will only present standard frequencies and not half-octaves).

1st comparaison : ________ Hz vs. __________ Hz , choice :   2nd comparaison : _______ Hz vs. __________ Hz, choice :   3rd comparaison : _________ Hz vs. __________ Hz, choice : <br><br>Predominant tinnitus frequency measured : _____________ Hz

4) In order to assess tinnitus loudness matching, First, measure the hearing threshold at the predominant tinnitus frequency measured previously and **record it on your evaluation sheet.** To measure the loudness of tinnitus, present the sound below the

auditory threshold and then Increase In steps of 1dB until the sound presented Is approximately equal to the sound of tinnitus. Repeat the procedure 2-3 times until you have a stable measurement of the sound. **Record each value measured on your evaluation sheet.**

Hearing threshold at the predominant tinnitus frequency : _____________ dB HL
1st loudness measure : _________ dB HL
2nd loudness measure : ________ dB HL
3rd loudness measure : __________ dB HL
Loudness of tinnitus (predominant frequency) : ______________ dB SL

5) Perform the minimum masking level measurement using white noise. First, measure the hearing threshold of white noise. **Record the hearing threshold value on your evaluation sheet.** To measure the minimum masking level, present the white noise at an infrathreshold level and then increase the level of the sound by 2-3 dB steps until the tinnitus is just masked. Repeat the procedure 2-3 times until you have a stable measurement of the sound. **Record each value of each response on your evaluation sheet.**

Hearing threshold of white noise : _____________ dB HL
1st masking measure: _________ dB HL
2nd masking measure : ________ dB HL
3rd masking measure: __________ dB HL
Miminum masking level (white noise) : ______________ dB SL

6) Perform the residual inhibition measurement. To do this, you will need to present a white noise sound10 dB above the minimum masking level obtained in step 5. Record this value on your evaluation sheet. You will have to present the noise at this level for 60 seconds and start a stopwatch once the sounds stops in order to record the duration of the inhibition. Ask the patient if their tinnitus has disappeared or changed after the

offset of the stimulation, and then ask them to tell you when the tinnitus returns to normal. **Record whether the residual inhibition was positive (gone or decreased) or negative (no change) and record the duration in seconds of the residual inhibition. Note any relevant comments, for example: tinnitus decreased slightly, changed frequency, etc**.

Level of white noise : _____________ dB HL
Residual Inhibition test result : Positive / Negative
Comments :
Duration of the Inhibition : ______________ seconds

Supplementary Material S2

Post laboratory questionnaire

Q1: During the tinnitus psychoacoustics lab, how did you experience being the clinician?

Q2: During the tinnitus psychoacoustics laboratory, how did you experience being the simulated patient?

Q3: Do you think these labs will help make you better future audiologists? **Yes / No**

If **yes** was your answer, what elements of these two labs will be most useful to you in your

future career as an audiologist and why?

If **no** was your answer, what improvements could achieve this goal?
